# Supplementary figures and images for: Nanoparticulate air pollution disrupts proteostasis in Caenorhabditis elegans
Source: PLoS One. 2023 Feb 23;18(2):e0275137. doi: 10.1371/journal.pone.0275137 (PMC9949623; doi:10.1371/journal.pone.0275137)

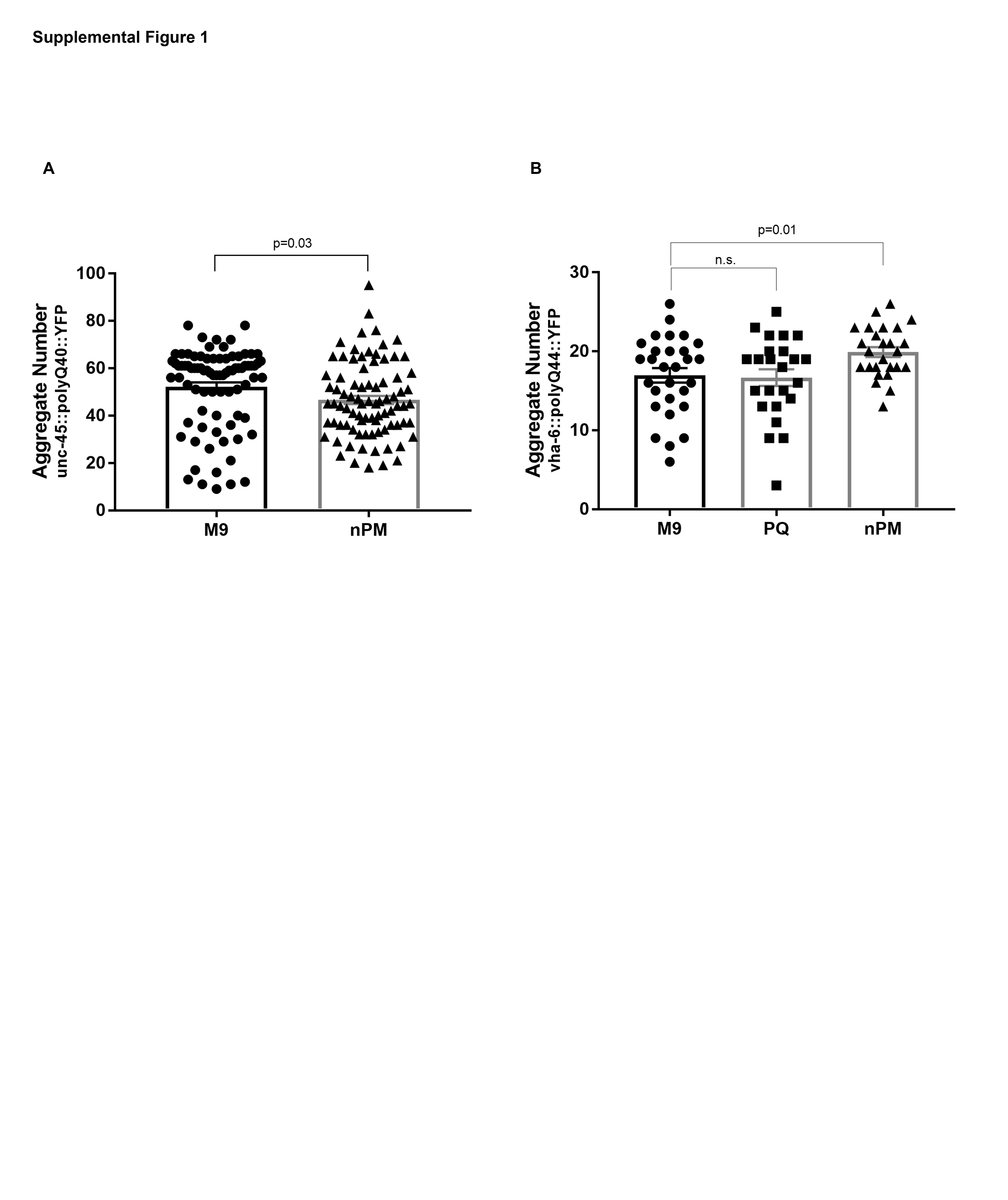

Supplement: S1 Fig — A) C. elegans expressing polyQ40::YFP in body wall muscle cells (strain AM141) were exposed for 3d to nanoparticulate matter (nPM), the oxidant paraquat (PQ), or mock exposed to vehicle (M9) starting at the L1 stage. Because L1 stage animals experienced low survival in PQ, they are omitted from this analysis. B) Animals expressing polyQ44::YFP in intestinal cells (strain OG412) were exposed to nPM for 72hrs starting at the L4 stage. After exposures, animals were allowed to recover for 3d on NGM plates seeded with OP50 at which time aggregate number was determined. Graphs depict the number of large visible aggregates in either body wall muscle cells (A) or intestinal cells (B). Bars represent the average number of aggregates with the number of aggregates in each individual also indicated (⚫, M9; ◼, PQ; ▲, nPM). Error bars represent the standard error of the mean (SEM). P-values are the results of T-tests with Welch’s correction. “n.s.” refers to differences that are not statistically significant. All exposures were performed in biological triplicate. (TIF) [file pone.0275137.s001.tif]

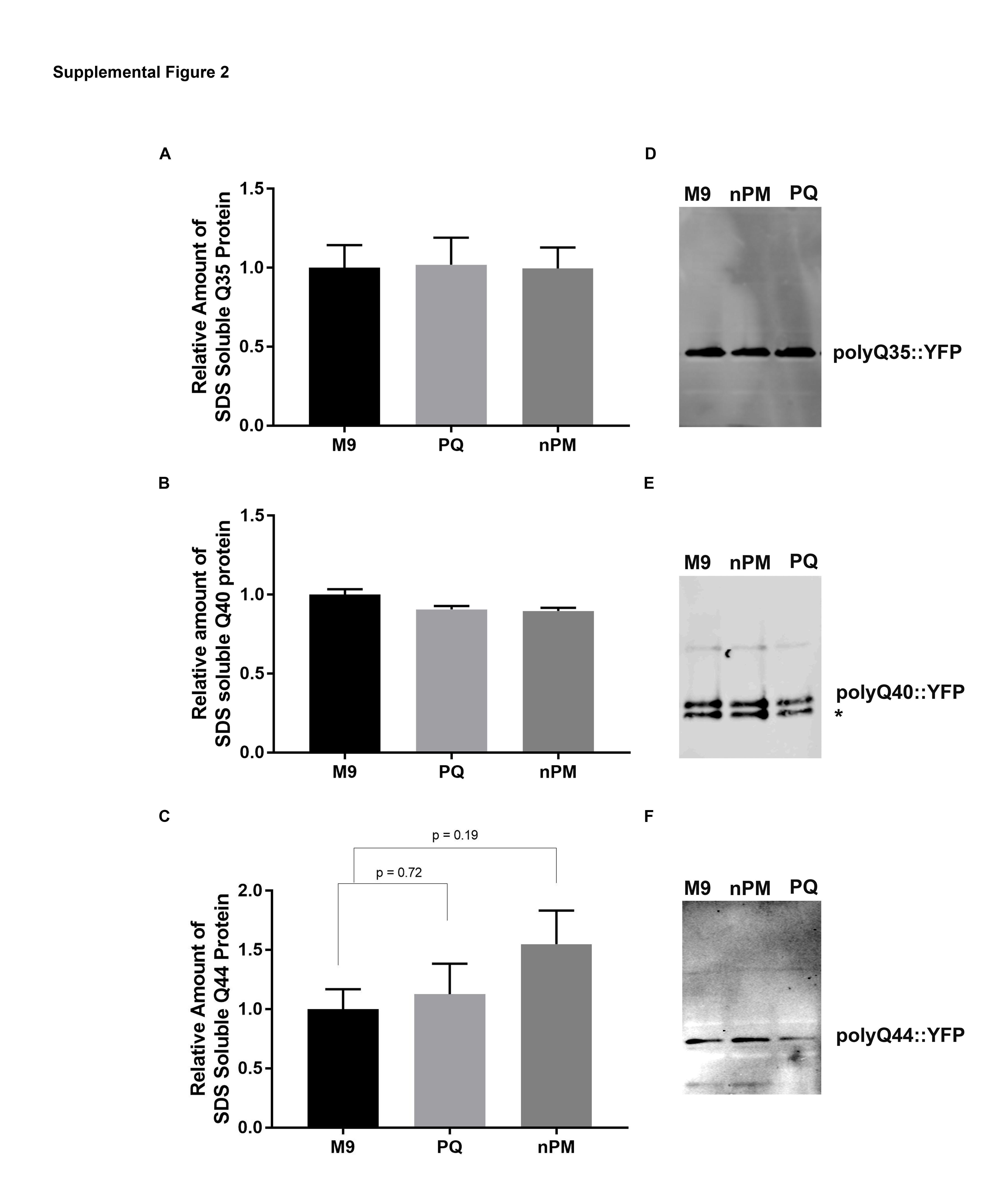

Supplement: S2 Fig — All animals were exposed for 3d to nanoparticulate matter (nPM), the oxidant paraquat (PQ), or mock exposed to vehicle (M9). Exposures started at the L4 stage for polyQ35::YFP animals (strain AM140) (A, D), at the L1 stage for polyQ40::YFP animals (strain AM141) (B, E), and at the L4 stage for the polyQ44::YFP animals (strain OG412) (C, F). Immunoblots of total protein were probed with an anti-GFP antibody. (A, B, C) Graphs represent the average amount of YFP-containing protein in biological triplicates. Error bars represent the standard error of the mean (SEM). All possible pairwise T-tests were performed with Welch’s correction and no statistically significant differences in steady-state protein levels were observed between treatments. P-values are only shown for polyQ44::YFP because that strain had the greatest amount of variability in western blot analysis. (D, E, F) Representative immunoblots. The doublet marked with a * likely represents YFP alone as the result of proteolysis between the polyQ40 and YFP moieties during sample preparation. Both upper and lower bands were included in the quantification. (TIF) [file pone.0275137.s002.tif]

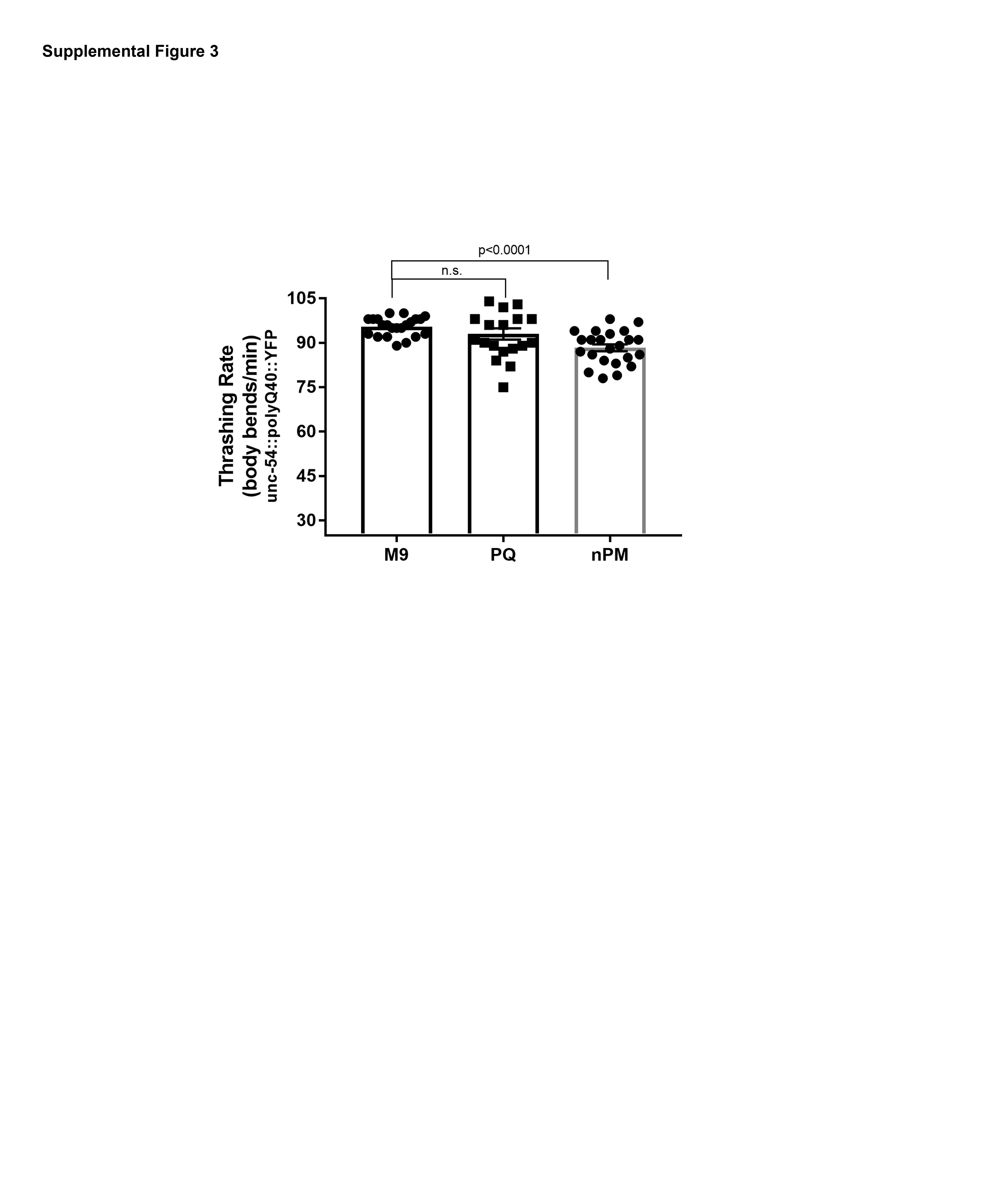

Supplement: S3 Fig — C. elegans expressing polyQ40::YFP in body wall muscle cells (strain AM141) were exposed for 3d to nanoparticulate matter (nPM), the oxidant paraquat (PQ), or mock exposed to vehicle (M9) starting at the L1 stage. Toxicity is represented as a function of thrashing rate in liquid measured 30min after exposure. Assays were performed in biological triplicate and the graphs represent the average thrashing rate with individual measurements indicated (⚫, M9; ◼, PQ; ▲, nPM). Error bars represent the standard error of the mean (SEM). P-values are the results of T-tests with Welch’s correction. “n.s.” refers to differences that are not statistically significant. (TIF) [file pone.0275137.s003.tif]

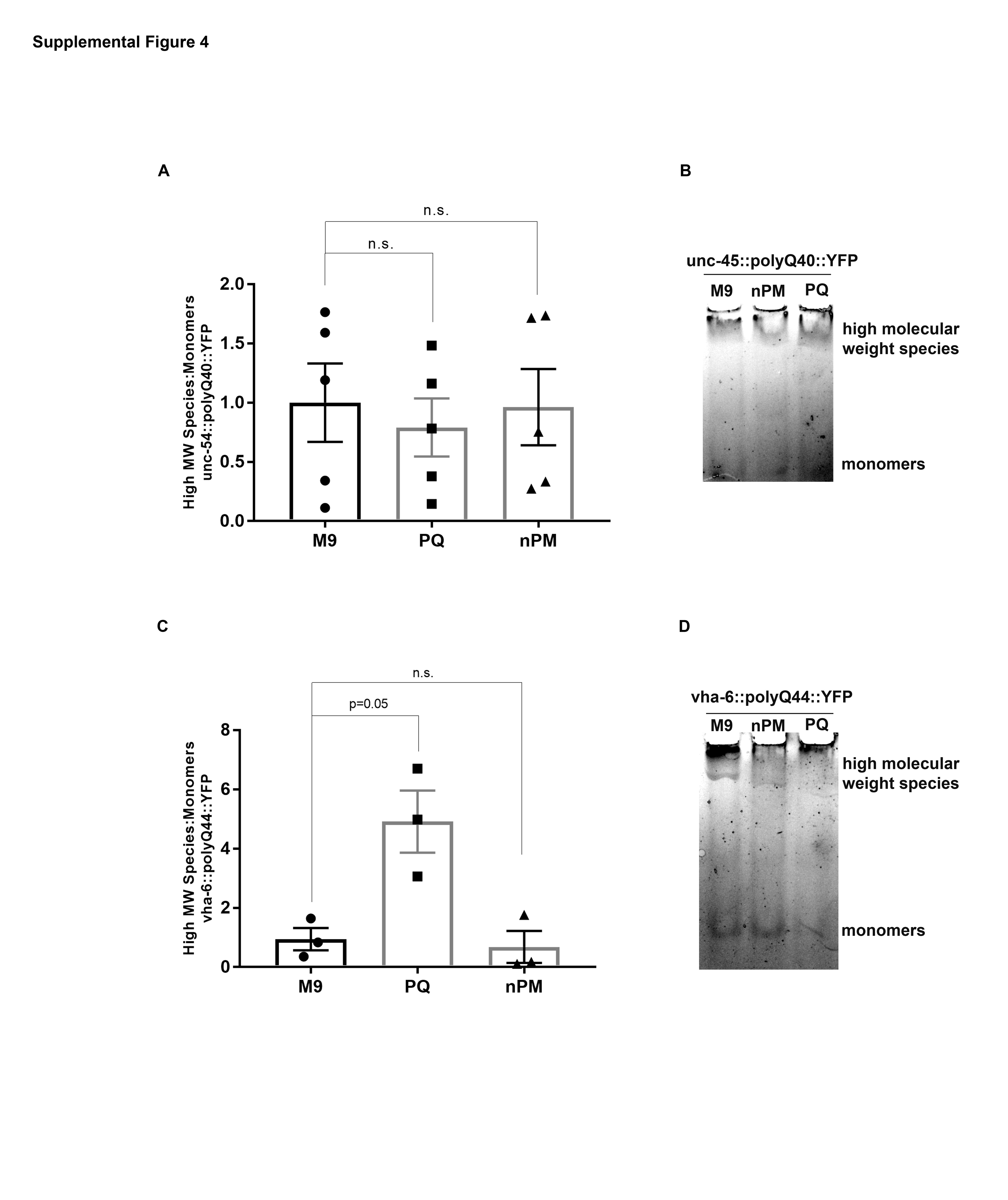

Supplement: S4 Fig — C. elegans were exposed for 3d to nanoparticulate matter (nPM), the oxidant paraquat (PQ), or mock exposed to vehicle (M9) starting at the L1 stage followed by 1hr recovery in the case of polyQ40::YFP (strain AM141) (A,B) or at the L4 stage followed by 3d of recovery for polyQ44::YFP animals (strain OG412) (C,D). Total native protein was extracted and native gel electrophoresis was performed in at least biological triplicate. A, C) The ratios of the indicated high molecular weight species to monomers within each lane are shown as averages of biological replicates (n = 5 for polyQ40::YFP and n = 3 for polyQ44::YFP) (bars) and also as individual biological replicates (⚫, M9; ◼, PQ; ▲, nPM). T-tests were performed with Welch’s correction. “n.s.” refers to differences that are not statistically significant. (B,D) Representative native gels showing in-gel YFP fluorescence with high molecular weight species and monomers indicated. (TIF) [file pone.0275137.s004.tif]
