# Supplementary material for: Nanoparticulate air pollution disrupts proteostasis in Caenorhabditis elegans
Source: PLoS One. 2023 Feb 23;18(2):e0275137. doi: 10.1371/journal.pone.0275137 (PMC9949623; doi:10.1371/journal.pone.0275137)

Original Image for Native Gel Shown in Fig. 3A

unc-54::Q35::YFP

M9 PQ nPM X X X

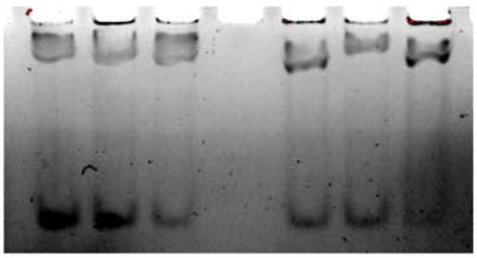

Original Images for Native Gels Shown in Sup. Fig. 4

Panel B:

unc-54::Q40::YFP

x x x M9 nPM PQ

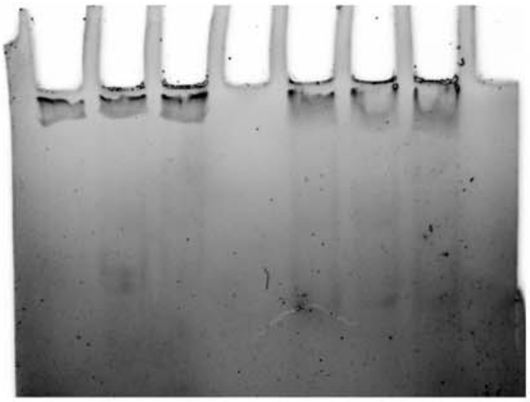

Panel D:

vha-6::polyQ44::YFP

x M9 nPM PQ x x

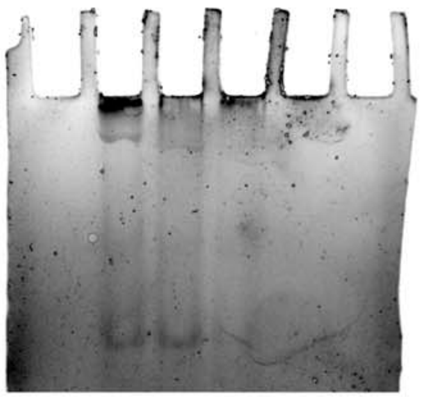

Original gel blot images for Supplmental Figure 2

Panel D:

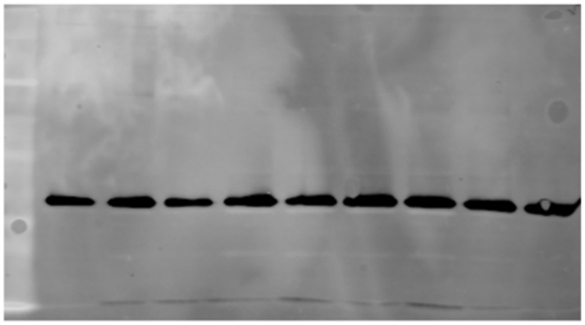

Panel E:

X X X X X X M9 nPM PQ

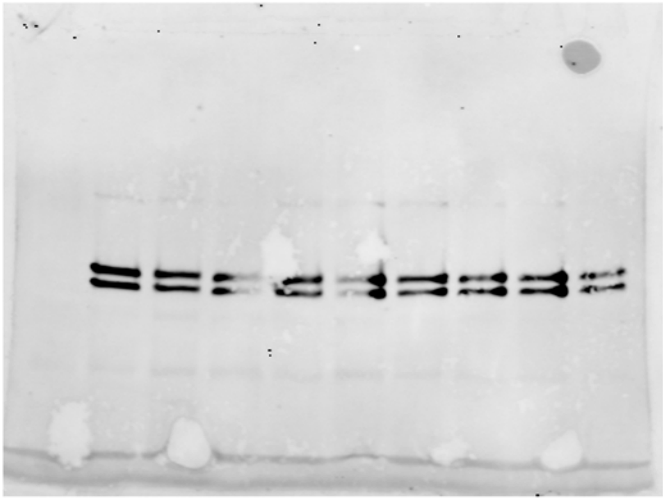

Panel F:

X X X X X M9 nPM PQ

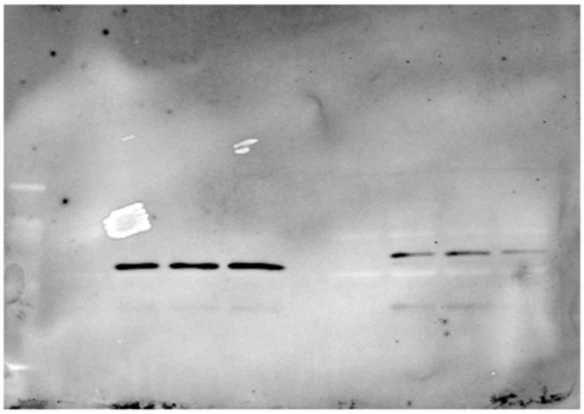

Supplement: S1 Raw images — (PDF) [file pone.0275137.s005.pdf]
