# Supplementary material for: Nanoparticulate air pollution disrupts proteostasis in Caenorhabditis elegans
Source: PLoS One. 2023 Feb 23;18(2):e0275137. doi: 10.1371/journal.pone.0275137 (PMC9949623; doi:10.1371/journal.pone.0275137)
Supplement: Attachment — Submitted filename: Response to reviewers.docx [file pone.0275137.s006.docx]

Dear Dr. Narayan,

Thank your for handling the peer review process for our manuscript (PONE-D-22-25226) titled “Nanoparticulate air pollution disrupts proteostasis in *Caenorhabditis elegans*.” We received reviews from two individuals (reviewer #1 and reviewer #2) and cannot thank them enough for their careful attention to this manuscript. All the comments were immensely helpful and thus we have nothing to rebut. Instead, we have addressed all the comments in our revised manuscript as described below. I hope that you and the reviewers agree that the revised manuscript is much improved.

Sincerely,

Dr. Elise Kikis, Ph.D.

Associate professor and chair of biology

The University of the South (Sewanee)

**Reviewer #1** recommended some “additional data and further analysis of the data to strengthen [our] conclusions”. These recommendations were immensely helpful and we have addressed all of them as described below.

1. **To better view the visual aggregates that were quantified in Figure 1B, include an inset with higher magnification that depicts the type of aggregate that was scored. Specifically, magnify the area at the end of the arrowheads.**

Figure 1B has been modified accordingly. Specifically, we put rectangles around the magnified areas and placed magnifications as insets. The reviewer was right that the resultant figures better portray the aggregates that were scored. The figure legend was modified accordingly.

2. **It is unclear what the reader should observe in Figure 1C. Addition of arrows to denote what the authors refer to as AB puncta and arrowheads to highlight what the authors refer to as larger aggregates is needed.**

To clarify what we refer to in Figure 1C as small puncta and larger aggregation, we did as the reviewer suggested and magnified a small region of each image and included an inset, similar to what we did for Figure 1B. We marked one small punctum with an arrow and one larger aggregate with an arrowhead in each inset as a way to illustrate what we are looking at. There are too many aggregates and small puncta to mark them all. The corresponding figure legend was modified accordingly.

3. **Supplemental Figure 2 shows the quantification of relative protein levels in each polyQ model. To support the conclusion that protein levels do not change, a representative gel image of the area quantified would be helpful. Specifically, polyglutamine tracts can lead to accumulation of proteins in the stacking gel of SDS PAGE gels. It would be important to note if protein accumulated in the stacking gel and if this was included in the final determination of protein levels. An image of a representative gel/blot to resolve this would be informative.**

Per the reviewer’s suggestions, we have now included representative western blot images in supplemental fig. 2 showing the bands that were quantified. The figure legend was modified accordingly. The polyQ40::YFP protein ran as a doublet as shown in Fig. S2E. We think that the lower band represents YFP-alone as the result of proteolysis during sample preparation. This has been indicated in the revised figure legend.

4. **In Supplemental Figure 2, the authors state that there is no statistical difference (text line 241 but do not provide p values. This is relevant for panel C as the relative level between M9 control and nPM look different.**

P-values for supplemental Fig 2C have been provided within the revised figure. The figure legend has alo been modified accordingly.

5. **Fix line 264. Change AB to polyQ**

Thank you for catching that! This should actually say “nPM”—not AB or polyQ. This has been corrected in the revised manuscript.

6. **The formatting in Figure 2B and 2C make it difficult to discern the impact of nPM on N2. It appears as though the lines overlap but a statement in the text would help resolve this confusion.**

There was no impact of nPM on N2, resulting in those lines/data points completely overlapping on the graph. This is an important point because it implies that nPM exerts its toxicity in a manner dependent on the presence of Abeta. This has been clarified in the text. Specifically, we added the following sentence to the results section. “It is noteworthy that, in contrast to the paralysis observed in Aβ animals, wild type (N2) animals remained mobile for the duration of the analysis regardless of the exposure paradigm. This implies that nPM is not toxic to wild type animals and further suggests that the observed paralysis in GMC101 is dependent on the presence of Aβ.”

7. **In Figure 2B, it is unclear if there is a statistically significant effect of the acute exposure to nPM for strain GMC101. There is an asterisk on the graph but it is unclear what this refers to. Furthermore, this reviewer questions if a t-test is the appropriate statistical analysis for this data set. The authors should clarify since Line 272 does not state if these differences (IT50) were statistically significant.**

The asterisks in Fig. 2B and Fig 2C represent the results of pair-wise t-tests for individual time points comparing +/-nPM. This has now been clarified in the figure legend. As the reviewer implies, T-test can tell us about each time point separately, but they do not allow us to determine statistically significant differences in the overall time course. This is why we also performed regression analysis and used the curves of best fit to calculated IT50s. This was implied in the methods section, but we have also clarified this point in the figure legend and corresponding results section. R^2^, SD, and p-values have been provided in the revised manuscript, and statistically significant differences are stated more explicitly.

8. **In Figure 3, it is unclear if the high molecular species are normalized to total protein in each lane or to control. Or are the values determined only by comparing intensity of the HMW species vs monomers for each strain. This reviewer recommends calculating relative amounts of HMW in total for each strain.**

The values were determined by comparing the intensity of the HMW species to monomers for each exposure paradigm for each strain. This allows us to see how each exposure alters the ratio (relative amounts) of HMW species compared to monomers. I thought this was clear in the results section, but we clarified it further in the methods section where we now indicate that the ratios were calculated separately for each lane. This is important because the ratios also serve as an internal control for inadvertent differences in loading.

9. **In Supplemental Figure 4, the figure legend states that protein was collected for the Q40 strain immediately instead of after 72 hours, which was the procedure for aggregate counting. Is there a rationale for why the treatment with nPM was not similar to the procedure for aggregate counting?**

The two procedures were the same. The 72hrs includes the exposure time in each case. “Immediate” was not the right word in Fig. S4, as there was a period of recovery on NGM plates following exposure. The legend has been revised to reflect this. There was also an error in the legend for Fig. S4 with respect to the polyQ44 recovery time. This was 3d following exposure, as described in the methods section on aggregate counting. We have revised the figure legend accordingly.

10. **Supplemental Figure 4 shows the quantification of high molecular weight species in the Q40 and Q44 models. A representative native gel image highlighting the area quantified would be beneficial.**

The representative gels have now been added.

Review #1 also offered three “additional suggestions to address within the text to further strengthen the manuscript.” We have addressed those suggestions as follows:

1. **Have the authors examined the impact of nPM on stress responses in PCD models? The authors demonstrate no impact on N2 animals but the extra challenge of expressing a misfolded disease protein along with the nPM may lead to activation of a stress response.**

The reviewer raises a good point that the combined challenge of misfolded protein and nPM could represent sufficient protein damage to trigger a transcriptional stress response. An alternative hypothesis is that nPM exacerbates protein misfolding by *not* triggering a (presumably cytoprotective) stress response. We have not directly addressed these hypotheses, but are things we are planning to look at. Because this manuscript represents a registered report that follows on a peer-reviewed and published registered protocol, we were limited in how much we could deviate from (or expand on) the proposed research plan. However, we have added the alternative hypothesis proposed by this reviewer to the end of the section on stress responses. Specifically, the revised manuscript now states, “It is possible that the combined challenge of nPM and an aggregation-prone disease-associated protein such as polyQ or Aβ may be sufficient to induce a stress response, whereas nPM alone is clearly insufficient. Alternatively, it may be that without robust cytoprotective stress responses, the buffering capacity of the proteostasis network remains low upon npM exposure, such that disease-associated protein aggregation/toxicity is exacerbated. These hypotheses can be tested in future studies by examining the effects of nPM on transcriptional stress responses in the polyQ35::YFP or polyQ40::YFP genetic backgrounds.”

2. **In Supplemental Figure 1A, nPM leads to an unexpected decrease in Q40 aggregates. To explain this surprising result, the authors state that Q35 is a “better sensor” than Q40. The authors should also consider that the treatments are done at different times in the developmental cycle of the worm, which may affect proteostasis demands.**

This is a good point. We have revised the text to now say, “These apparently contradictory results can likely be explained by exposures being performed at different times in the developmental or cycle of the animal or by polyQ35::YFP being a better sensor than polyQ40::YFP to detect subtle changes in the protein folding environment.”

3. **In the conclusions section, it would be helpful if the authors offer their insight into why nPM that exacerbates protein misfolding in body wall muscles can suggest deficiencies in protein folding of AB species in neurons leading to dementia.**

This question is especially important as it seems to get at the relevance of this study, done in *C. elegans* muscle cells, to a human neurodegenerative disease. It is similar to a point also made by reviewer #2 (see below). In terms of human disease, at least two hypotheses exist for the mechanism of action of nPM as an environmental risk factor for AD. The first is that nPM triggers neuroinflammation, which in turn results in proteostasis disruption. The second is that nPM directly disrupts proteostasis independently from neuroinflammation. While these two hypotheses are not mutually exclusive, we wanted to know whether the second hypothesis (direct effect of nPM on proteostasis) is possible. *C. elegans* is a powerful system to address this question, as it lacks an inflammatory response, allowing us to uncouple the two processes, and polyQ/ABeta are established sensors of proteostasis decline. We have elaborated on these points in the revised “conclusions” section.

We also further discussed what direct nPM exposure might look like in humans. Specifically, we noted that, “Because nPM disrupts proteostasis in *C. elegans*, it seems theoretically plausible that nPM exposure may worsen Alzheimer's disease symptoms and age-related dementia, at least in part by overwhelming the proteostasis network and thereby disrupting the delicate proteostasis balance. This may be especially true for olfactory neurons, which are in direct contact with the air and which experience pathological protein misfolding early in Alzheimer’s’ disease progression.”

**Review #2** offered one comment and one suggestion. The comment was that, **“one short-coming of the study is uncertainty regarding the potential exposure in vulnerable neurons of human brain relative to the dose of nanoparticulates that the worms were exposed to.”**

We cannot say much about dosing because we don’t know how much nPM is getting into the animals in our study or how dosing in *C. elegans* over a short time period would correspond to chronic nPM exposure for people spending lifetimes in areas with poor air quality. However, as review #1 also asked about the relevance of the *C. elegans* model to the human brain, we did elaborate on this in the concluding paragraph as described above (response to reviewer #1, additional suggestion #3).

Reviewer #2’s suggestion was that **“that in Fig 1C, the authors should indicate the number of replicates that were examined to produce the representative images.”**

Good point. To address this, we have added the following sentence to the Fig. 1 legend, “Exposures were performed in biological triplicate, with ~20 animals examined per exposure per triplicate.”
